# Essential Oil Quality in Medicinal and Aromatic Plants: Linking Plant Biosynthesis, Matrix Selection and Extraction Strategy

**DOI:** 10.3390/plants15182771

**Published:** 2026-09-10

**Authors:** Oumayma Ben Youchret-Zallez, Renato Spigarelli, Ahmed Kouki, Lina Mbirki, Mossadok Ben-Attia, Alberto Bernacchi, Maria Chiara Valerii, Enzo Spisni

**Affiliations:** 1 Laboratory of Chemical Materials LR13ES08, Faculty of Sciences of Bizerte, University of Carthage, Zarzouna, Bizerte 7021, Tunisia; oumayma.benyouchret@fsb.ucar.tn; 2 Department of Biological, Geological, and Environmental Sciences (BiGeA), University of Bologna, Via Selmi 3, 40126 Bologna, Italy; renato.spigarelli2@unibo.it (R.S.); enzo.spisni@unibo.it (E.S.); 3 Environment Biomonitoring Laboratory (LR01/ES14), Faculty of Sciences of Bizerte, University of Carthage, Zarzouna, Bizerte 7021, Tunisia; ahmed.kouki@fsb.ucar.tn (A.K.); mossadok.benattia@fsb.ucar.tn (M.B.-A.); 4 Laboratory of Integrated Physiology, Faculty of Sciences of Bizerte, University of Carthage, Zarzouna, Bizerte 7021, Tunisia; linambirki@gmail.com

**Keywords:** essential oils, medicinal and aromatic plants, phytochemistry, plant matrix, biosynthetic pathways, extraction technologies, green extraction, essential oil quality

## Abstract

Essential oils (EOs) are complex mixtures of volatile secondary metabolites produced by medicinal and aromatic plants (MAPs), mainly including terpenes, terpenoids and phenylpropanoid/benzenoid derivatives. Their composition depends on the plant species, chemotype, organ, developmental stage, environmental conditions and post-harvest processing, and directly affects EO yield, EO profile, biological properties, standardization and industrial value. This narrative and conceptual review connects plant biosynthesis, environmental regulation, matrix selection and extraction strategy as interdependent determinants of EO quality. Terpenoid constituents originate mainly from the mevalonate (MVA) and methylerythritol phosphate (MEP) pathways, whereas phenylpropanoid and benzenoid volatiles derive largely from the shikimate/phenylalanine pathway. Conventional extraction methods, such as hydrodistillation and steam distillation, remain widely used, while intensified and complementary approaches, including microwave-assisted methods, ultrasound pretreatment, supercritical CO_2_ extraction and solvent-free methods, are increasingly investigated to improve selectivity and reduce extraction time, solvent use, water demand and energy consumption. Rather than ranking extraction methods only by yield, this review emphasizes that EO quality should be interpreted through an integrated plant-to-extraction framework, in which botanical raw material, target chemical classes, matrix properties, compound stability and intended application jointly guide the selection of an extraction strategy. This integrated perspective may support more reproducible, application-oriented and sustainable EO production.

## 1. Introduction

Essential oils (EOs) are complex mixtures of volatile and semi-volatile compounds produced by medicinal and aromatic plants (MAPs). They are generally hydrophobic, oily and strongly odoriferous fractions obtained from different plant organs, including leaves, flowers, stems, roots, bark, seeds and fruits. Their chemical composition is mainly represented by terpenes, terpenoids, phenylpropanoid/benzenoid derivatives and other aroma-related secondary metabolites, which contribute to the characteristic odor, biological properties and industrial value of each oil [1,2,3]. Several EO constituents have been investigated for antimicrobial, antioxidant, anti-inflammatory and anticancer activities, further supporting the relevance of EO chemical composition for targeted applications [1,4,5].

The composition and yield of EOs are highly variable and depend on the interaction between botanical, physiological, environmental and technological factors. The plant species, genotype, chemotype, plant organ, developmental stage, cultivation area, mineral nutrition, climatic conditions, harvest time and post-harvest handling may all influence EO yield and the relative abundance of key volatile constituents. For this reason, EO quality cannot be defined only by botanical identity, but requires an integrated evaluation of the plant raw material, its biosynthetic capacity and the environmental conditions under which it is produced. This variability is particularly relevant for industrial applications, because changes in EO composition may affect aroma, biological activity, safety, reproducibility and commercial standardization [6,7,8].

Beyond its general industrial relevance, compositional variability is particularly important because EO quality is increasingly evaluated not only by total oil yield, but also by the relative abundance of specific marker or target constituents. Quality specifications for commercial EOs commonly rely on botanical identity, chromatographic profiling and compositional ranges for major compounds, as illustrated by sage oils, for which International Organization for Standardization (ISO) standards define ranges for constituents such as 1,8-cineole, thujones and camphor [9]. At the same time, EO constituents are relevant across food, cosmetic and pharmaceutical applications, where sensory properties, antimicrobial activity, safety and bioactivity may be associated with defined chemical markers or selected volatile constituents [10]. In food-related research, individual constituents such as carvacrol, thymol, eugenol, cinnamaldehyde, linalool, limonene and 1,8-cineole are frequently investigated in relation to antimicrobial mechanisms, food preservation and interactions with food matrices [11,12,13]. Therefore, target-compound abundance should be considered a central criterion for EO standardization and application-oriented extraction strategies.

From a biochemical point of view, most EO constituents originate from a limited number of biosynthetic routes. Terpenes and terpenoids derive from isoprenoid metabolism through the mevalonate (MVA) and methylerythritol phosphate (MEP) pathways, whereas phenylpropanoid and benzenoid volatiles originate mainly from the shikimate/phenylalanine pathway. These pathways provide the biochemical basis for EO chemotype formation and help explain why the genotype, environmental stress, developmental stage and plant organ can modify the final volatile profile. Understanding the biosynthetic origin of EO constituents is therefore essential for linking plant physiology with EO quality and extraction behavior [2,14,15].

Extraction is a decisive step in EO production because it determines which compounds are selectively recovered, enriched or lost in the final product and, under conditions promoting hydrolysis, oxidation, isomerization or thermal degradation, which constituents may undergo true chemical modification. Conventional techniques, such as hydrodistillation and steam distillation, remain widely used because they are simple, solvent-free and scalable. However, prolonged heating and water exposure may alter thermolabile or highly volatile constituents. Emerging, intensified and complementary approaches, including microwave-assisted methods, ultrasound pretreatment, supercritical CO_2_ extraction and solvent-free approaches, are increasingly investigated because they may reduce extraction time, energy demand, water or solvent consumption and waste generation, while improving selectivity and preservation of target compounds. Nevertheless, each technique has specific advantages and limitations, and no single extraction method can be considered universally optimal for all botanical matrices or applications [3,8].

Representative previous reviews have focused on extraction, separation and purification methods for plant EOs [3]. Other contributions have examined the extraction and analytical characterization of plant-derived oils more broadly [8], EOs as multicomponent mixtures and their health-related effects [10], or antimicrobial mechanisms and potential food applications [13]. To address the comparatively limited integration of these areas, the present review connects plant biosynthetic origin, environmental regulation, organ- and matrix-dependent volatile localization and extraction-dependent selectivity within a single quality-oriented plant-to-extraction framework. In this framework, EO quality is interpreted as the outcome of biological, environmental, chemical and technological determinants acting together before and during extraction. Compound stability, product boundaries, standardization, sustainability and intended application then provide the basis for a decision-oriented comparison of extraction technologies.

### 1.1. Literature Search Strategy and Selection Criteria

This article was designed as a narrative and conceptual review rather than as a systematic review or meta-analysis. A targeted literature search was conducted to identify representative studies and reviews relevant to essential oil quality, plant biosynthesis, medicinal and aromatic plants, plant-matrix effects and extraction technologies. Searches were performed in PubMed, Google Scholar and major publisher platforms, including ScienceDirect, SpringerLink, Wiley Online Library and MDPI, and were complemented by citation screening of key papers and relevant references identified during manuscript preparation. The literature search focused mainly on publications from the last ten years and was last updated in June 2026, while older references were retained when they represented foundational or directly relevant sources for established biochemical pathways, extraction principles, biological activity or analytical concepts.

Search strings combined terms related to the product category (“essential oil”, “volatile oil”, “volatile profile”, “aromatic extract”), plant source and biosynthesis (“medicinal and aromatic plants”, “plant matrix”, “chemotype”, “mevalonate pathway”, “MVA pathway”, “MEP pathway”, “phenylpropanoid”, “shikimate pathway”), extraction and analytical approaches (“hydrodistillation”, “steam distillation”, “microwave-assisted extraction”, “solvent-free microwave extraction”, “ultrasound-assisted extraction”, “supercritical CO_2_ extraction”, “solvent extraction”, “headspace”, “HS-SPME”), and quality or sustainability aspects (“GC–MS”, “GC–FID”, “quality control”, “standardization”, “authenticity”, “green extraction”, “energy consumption”, “sustainability”). English-language peer-reviewed articles, reviews and standards-oriented sources were prioritized. Studies were considered particularly relevant when they linked botanical material, chemical composition, extraction conditions, EO yield, volatile profile, quality control or sustainability-related indicators.

Comparative examples included in the comparative synthesis presented later in Section 4 were selected when they provided within-study comparisons between extraction or analytical approaches applied to the same plant matrix, or when they directly illustrated method-dependent effects on EO yield, volatile composition, extraction time, energy demand, water or solvent use, or analytical profiling. Studies were excluded from comparative synthesis when the plant material, extraction basis, yield denominator, analytical method or reported outcomes were insufficiently described for meaningful interpretation within the scope of this review.

### 1.2. Product Terminology and Scope

In this review, the term essential oil (EO) denotes a plant-derived volatile product obtained mainly by hydrodistillation, steam distillation or mechanical expression, particularly from citrus peels. By contrast, products obtained by organic solvent extraction are referred to as EO-related aromatic extracts rather than as classical EOs. These include concretes, absolutes, resinoids and oleoresins, which may contain volatile fragrance constituents together with waxes, pigments, resins or other non-volatile lipophilic compounds. The unqualified term extract is used only when the product cannot be identified more specifically from the cited study or when different non-distilled products are discussed collectively. Hydrolates, or aromatic waters, are considered aqueous distillation co-products containing water-soluble volatile constituents.

Supercritical CO_2_ extraction (SFE-CO_2_) may produce volatile-rich fractions, but depending on extraction conditions it can also recover non-volatile lipophilic material; therefore, SFE-CO_2_ products are described here as CO_2_ extracts or volatile-rich fractions unless the cited study clearly refers to an EO-like product. Throughout this review, EO profile refers to the reported composition of a recovered EO, whereas volatile profile denotes the set and relative distribution of volatile compounds detected in plant material, headspace samples or non-EO products under the stated analytical conditions. Headspace and HS-SPME analyses are treated as analytical volatile-profiling techniques, not as productive extraction methods.

**Figure 1:**
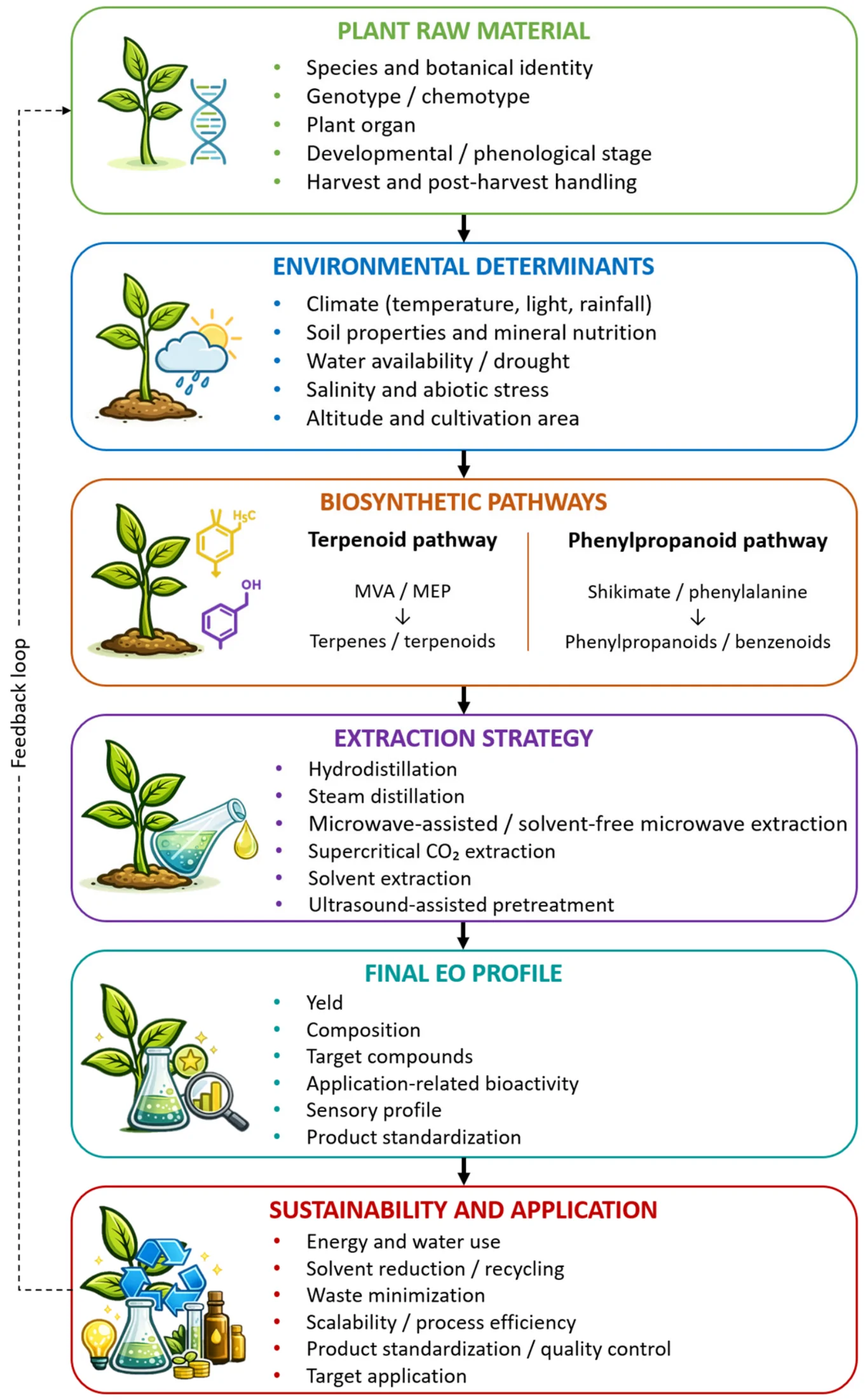
Integrated conceptual framework of essential oil quality and sustainability. The scheme represents an interacting plant-to-extraction system rather than a strictly linear causal chain. Plant raw material, environmental determinants, post-harvest handling, biosynthetic pathways and extraction parameters jointly shape the final EO profile and quality-related endpoints, including yield, composition, target compounds, application-related bioactivity, sensory profile and product standardization. The dashed feedback arrow indicates that sustainability and application-oriented requirements can inform raw-material selection, cultivation and post-harvest choices, extraction strategy and process optimization. Abbreviations: EO, essential oil; MEP, methylerythritol phosphate; MVA, mevalonate.

Accordingly, this review adopts an integrated plant-to-extraction framework linking plant biosynthesis, environmental determinants and extraction technologies as interdependent factors shaping EO yield, composition, quality, standardization and sustainability. EO quality arises from a continuum of determinants acting before and during extraction. The botanical identity, cultivar and cultivation environment define the biological potential of the raw material, whereas the selected plant organ determines the anatomical and chemical matrix from which volatiles must be released. The biosynthetic origin of EO constituents influences their volatility, polarity and thermal sensitivity. Extraction then determines which compounds are selectively recovered, enriched or lost and, under conditions promoting hydrolysis, oxidation, isomerization or thermal degradation, which constituents may undergo true chemical modification. Together, these factors shape the final EO yield, EO profile, application-related bioactivity and standardization. The conceptual framework linking plant raw material, environmental regulation, biosynthetic pathways, extraction strategy and final EO quality is summarized in Figure 1.

## 2. From Plant Identity to Plant Matrix: Pre-Extraction Determinants and Implications for Essential Oil Quality

Essential oil quality begins to be shaped before extraction, at the level of the plant raw material. Within the plant-to-oil continuum, botanical identity, cultivar or chemotype, cultivation environment and plant organ represent key pre-extraction determinants. Botanical identity defines the biosynthetic potential of the species or cultivar; environmental conditions and abiotic stresses modulate biomass production, EO yield and the relative abundance of volatile constituents; and the plant organ selected determines the anatomical and chemical matrix from which volatiles must be released. Therefore, this section focuses on how plant identity, environmental regulation and matrix selection influence EO quality before extraction. These pre-extraction characteristics also affect extraction efficiency, reproducibility and resource demand.

### 2.1. Medicinal and Aromatic Plants as Chemically Variable Raw Materials

In essential oil production, medicinal and aromatic plants should not be considered only as botanical sources of volatile compounds, but as chemically variable raw materials whose quality depends on multiple biological and environmental determinants. Botanical identity is therefore only the first level of raw-material characterization. For industrial and research purposes, EO quality must also be defined by EO yield, chemotype stability, the plant organ used, the developmental or phenological stage at harvest, and the relative abundance of target volatile constituents [16]. This variability is particularly relevant because the same species, or even the same cultivar, may produce EOs with different yields and EO profiles when grown under different soil, climatic or agroecological conditions [17,18,19]. Thus, EO quality should be interpreted as a dynamic property of the plant raw material, resulting from the interaction between the genotype, organ, developmental stage, soil properties and cultivation environment, rather than as an invariant characteristic of a given plant species [6,16,19]. Chrysargyris et al. (2020) [19] showed that the same altitude-related cultivation contrast produced opposite effects depending on the species: mountainous conditions increased EO yield in *Artemisia abrotanum* and *Laurus nobilis*, but decreased it in *Pelargonium radens* (*syn. Pelargonium roseum*), *Mentha spicata*, *Lavandula angustifolia* and *Aloysia citrodora* (*syn. Aloysia triphylla*). In *Artemisia abrotanum*, altitude shifted the EO profile toward higher proportions of 1,8-cineole and p-cymene in mountain-grown plants, while reducing camphor and camphene compared with plants grown in plain conditions, confirming that environmental conditions affect not only EO productivity but also the EO profile. These findings indicate that environmental conditions modulate EO production in a species-dependent manner and affect both yield and chemical composition.

Several studies specifically demonstrated that EO yield and composition may vary when the same species or cultivar is grown in different agroclimatic areas. In *Curcuma longa* cv. Lakadong, plants cultivated across five agroclimatic regions of Odisha showed significant differences in both EO yield and the gas chromatography–mass spectrometry (GC–MS) profiles of leaf and rhizome EOs; α-phellandrene in leaf oil varied from 31.78 to 39.89%, whereas ar-turmerone in rhizome oil varied from 36.18 to 44.36%. Temperature, humidity and altitude were identified as major environmental factors affecting EO quality and yield [18].

Similarly, the high-yielding turmeric cultivar *Curcuma longa* cv. Roma showed wide variation in leaf and rhizome EO yield and composition across nine agroclimatic zones. Leaf oil yield ranged from 0.37 to 1.00%, rhizome oil yield from 0.39 to 0.70%, α-phellandrene in leaf oil from 39.0 to 82.35%, and turmerone in rhizome oil from 12.6 to 71.0%. These findings indicate that soil nutrients and environmental factors strongly affect EO production and quality [17].

Comparable evidence has also been reported in other MAPs. Varieties of *Mentha* × *piperita* cultivated at two altitudes, 1100 and 1650 m above sea level, on identical soils showed that EO accumulation was highly dependent on climatic conditions, whereas the volatile composition varied only slightly within each variety but differed markedly among varieties [20]. In a comparative study of eight MAP species grown under mountainous and plain conditions in Cyprus, the cultivation environment affected EO yield, chemical composition and antioxidant activity for most species; the authors concluded that altitude-related environmental conditions can be used to select specific ecosystems for producing high-value MAP-derived products [19].

These findings indicate that the geographical origin and cultivation environment are critical determinants of EO quality and standardization. Therefore, the selection of plant raw material for EO production should consider not only species identity, but also cultivar, cultivation area, soil characteristics, harvest stage and target chemotype. This aspect is particularly relevant for sustainable EO production, because raw materials yielding predictable EO profiles may contribute to improving extraction efficiency, limiting unnecessary processing steps, and supporting reproducible quality for food, cosmetic and pharmaceutical applications [16,17,18,19].

### 2.2. Environmental Stress as a Determinant of EO Productivity and Chemotype

Environmental stress represents a specific level of raw-material modulation because it can alter both plant productivity and the metabolic allocation toward secondary compounds. Abiotic factors such as drought, salinity, temperature, light, heavy metals and soil nutrient availability may affect growth, photosynthesis, biomass accumulation and secondary metabolite biosynthesis in medicinal and aromatic plants [6]. Importantly, these factors do not act according to a single universal rule: the direction and magnitude of the response depend on the species, genotype, stress intensity, duration and compound class. For instance, Mahajan et al. (2020) [6] reported that salinity can reduce essential oil yield in several MAPs, including *Mentha* × *piperita*, *Mentha pulegium*, *Thymus maroccanus*, *Ocimum basilicum*, *Melissa officinalis*, *Origanum majorana* (*syn. Majorana hortensis*), *Matricaria chamomilla* and *Salvia officinalis*, whereas low levels of salt stress may increase EO percentage in species such as *Satureja hortensis*, *Salvia officinalis* and *Thymus vulgaris*. Similarly, temperature stress can modify secondary metabolite accumulation: elevated temperature has been associated with increased ginsenoside accumulation in *Panax* spp. and enhanced terpene emission, whereas cold stress may promote anthocyanin accumulation and artemisinin biosynthesis in *Artemisia annua* [6]. In EO-producing plants, these stress-induced metabolic changes are particularly relevant because they may reshape the volatile profile and, consequently, chemotype, biological activity and commercial standardization. In Mediterranean *Salvia* spp., Karalija et al. (2022) highlighted that climate-related stressors, including increasing soil salinity, aridity and temperature changes, may seriously affect the commercial production of sage EOs [9]. In *Salvia officinalis*, soil salinity markedly changed the dominant EO constituents: non-stressed plants were characterized mainly by viridiflorol, low salinity increased the relative proportion of viridiflorol, moderate NaCl stress shifted the profile toward 1,8-cineole and α-thujone, whereas higher salinity strongly increased manool. This example shows that salinity may not only affect EO yield, but also redirect the dominant EO profile. Temperature can also influence EO composition: in *S. officinalis* subsp. *lavandulifolia*, elevated growing temperatures above 24 °C and up to 32 °C were associated with a decrease in monoterpenes and an increase in sesquiterpenes, with changes in β-caryophyllene, caryophyllene oxide and manool, potentially leading to chemotype variation [9]. Together, these examples indicate that environmental stressors should not be interpreted only as agronomic constraints, but also as modulators of EO quality. However, because the same stress may increase, decrease or leave unchanged EO yield and individual volatile constituents depending on the plant system, environmental stress must be managed according to the target chemotype, desired biological activity and final application. Among these abiotic stresses, drought is particularly relevant for sustainable EO production because it directly affects irrigation requirements, water-use efficiency, primary productivity and secondary metabolism [21,22,23].

Water deficit generally reduces plant growth, photosynthetic performance, biomass accumulation and crop yield, but its effects on EO content, EO yield and volatile composition are species-, genotype- and stress-intensity-dependent. For example, moderate drought may increase the concentration of some secondary metabolites, whereas severe or prolonged drought may reduce biomass and therefore decrease the total EO yield per plant or per cultivated area [21,22,23].

Experimental studies show that drought may affect productivity and EO-related traits in different ways. In six Lamiaceae species subjected to deficit irrigation, drought reduced fresh biomass in *Lavandula latifolia*, *Mentha* × *piperita* and *Thymbra capitata* (*syn. Thymus capitatus*), while fresh biomass remained unchanged in *Salvia lavandulifolia*, *Salvia sclarea* and *Thymus mastichina*; EO content was reduced in *L. latifolia* and *S. sclarea*, whereas other species showed no significant changes in EO content under the imposed water deficit [22]. In *Ocimum* species, severe drought stress reduced relative water content, plant size, leaf area, fresh and dry herb yield, EO content and EO yield, while the major EO compounds remained unchanged regardless of soil water capacity, indicating that drought may strongly affect productivity without necessarily altering the dominant EO chemotype [23].

Drought can also modify the concentration of individual volatile constituents. In *Mentha* × *piperita*, moderate and severe drought increased total EO yield compared with regularly watered plants, and specific major EO constituents such as menthone, menthol and pulegone increased under drought stress or treatments with plant growth-promoting rhizobacteria (PGPR), whereas limonene and menthofuran were not significantly modified; at the same time, volatile organic compound (VOC) emissions decreased under drought, especially under severe stress [24]. In *Thymus vulgaris*, prolonged water deficit altered the volatile profile differently in drought-sensitive and drought-tolerant cultivars, with α-phellandrene, o-cymene, γ-terpinene and β-caryophyllene identified among the most relevant compounds involved in drought-stress adaptation [25].

These findings show that drought can affect both general productivity and individual EO constituents, although the two effects do not necessarily co-occur. In some cases, drought primarily reduces biomass and total EO yield; in others, it changes EO content, VOC emissions or the relative abundance of specific monoterpenes and sesquiterpenes. Consequently, the best cultivation condition should not be defined only by maximum plant growth, but by the balance among biomass productivity, EO yield, target compound concentration, water-use efficiency and chemical reproducibility [21,22,24,25].

From a sustainable extraction perspective, environmental stress is relevant because it can modify the chemical nature of the raw material before extraction. Changes in the relative abundance of monoterpenes, sesquiterpenes, oxygenated terpenoids or phenylpropanoids may influence volatility, polarity, thermal sensitivity and biological activity of the EO fraction. Therefore, raw materials produced under different environmental conditions may require adjusted extraction parameters to preserve target compounds and obtain reproducible EO quality. Integrating environmental control, chemotype monitoring and sustainable extraction technologies is thus essential for improving EO standardization while reducing water, energy and processing inputs [6,9,19].

### 2.3. Plant Matrix and Extraction Method as Joint Determinants of EO Quality

After species identity and cultivation conditions have been considered, sustainable EO extraction depends mainly on the plant matrix selected and on the extraction technology applied. A matrix-oriented approach is more informative than a purely botanical classification because leaves, flowers, fruits, seeds, bark, roots and rhizomes differ in anatomical organization, volatile localization, moisture content, matrix compactness and susceptibility to thermal, hydrolytic or oxidative changes. Consequently, the same extraction method may not be equally suitable for different plant organs, and different extraction methods applied to the same plant material may generate EOs with different yields, EO profiles, sensory properties, biological activity, extraction time, energy demand and process feasibility. Thus, plant matrix, target chemical class and intended application should jointly guide the choice of extraction technology [3,26].

Comparative studies show that different technologies applied to the same plant material may produce oils that differ in composition, sensory quality, biological activity and process-related indicators. In *Lavandula* × *intermedia* ‘Margaret Roberts’, steam distillation, hydrodistillation and cellulase-assisted hydrodistillation differed in extraction time, energy consumption, camphor content, odor quality, scale-up considerations and economic feasibility, showing that method selection depends not only on EO yield but also on quality and process constraints [27]. In three *Curcuma* species, hydrodistillation and solvent-free microwave extraction produced oils with qualitatively similar major compounds but significant quantitative differences; hydrodistilled oils were dominated by hydrocarbon compounds, whereas solvent-free microwave-extracted oils were dominated by oxygenated compounds and showed improved extraction efficiency and biological activities [28]. Similarly, comparison of hydrodistillation and steam distillation in *Ferulago contracta*, *Salvia rosmarinus* (*syn. Rosmarinus officinalis*) and *Lavandula sublepidota* showed that the type of distillation affected EO yield, chemical composition, total phenolic content, total flavonoid content and antioxidant activity [26].

Accordingly, MAP classification should be used as a decision-making framework only when it integrates botanical identity with matrix-oriented and extraction-oriented variables. Information on the species or cultivar, plant organ, tissue structure, moisture content, active constituent class, target compounds and intended application can guide the choice between conventional distillation, microwave-assisted hydrodistillation, solvent-free microwave extraction, ultrasound-assisted extraction, enzyme-assisted extraction, supercritical CO_2_ extraction or other emerging technologies. This approach shifts MAP classification from a static botanical list to a functional tool for optimizing EO yield, preserving target compounds, reducing unnecessary water and energy use, limiting degradation and supporting reproducible EO quality [3,26,27,28].

The matrix-oriented approach is particularly important when the industrial target is a specific volatile constituent rather than total EO yield. For example, β-caryophyllene is widespread among EO-producing taxa, but its relative abundance can vary markedly among organs of the same species. In *Syzygium cumini*, trans-β-caryophyllene represented 1.35% of leaf EO, 13.6% of fruit EO, 60.79% of seed EO and 0.34% of bark EO, showing that plant-organ selection can be decisive for obtaining a β-caryophyllene-rich oil [29]. Similarly, in *Synedrella nodiflora*, (E)-β-caryophyllene was reported at 11.7–22.2% in root EO, 7.1–9.0% in stem EO and 20.0–30.3% in leaf EO, further supporting the need to match target compounds with the most appropriate plant matrix [30]. Overall, these data suggest that EO production requires an integrated framework that connects plant identity, environmental conditions, plant matrix and extraction technology, rather than treating botanical classification, cultivation and extraction as separate steps.

## 3. Biosynthetic Origin and Chemical Classes of Essential Oil Constituents

**Figure 2:**
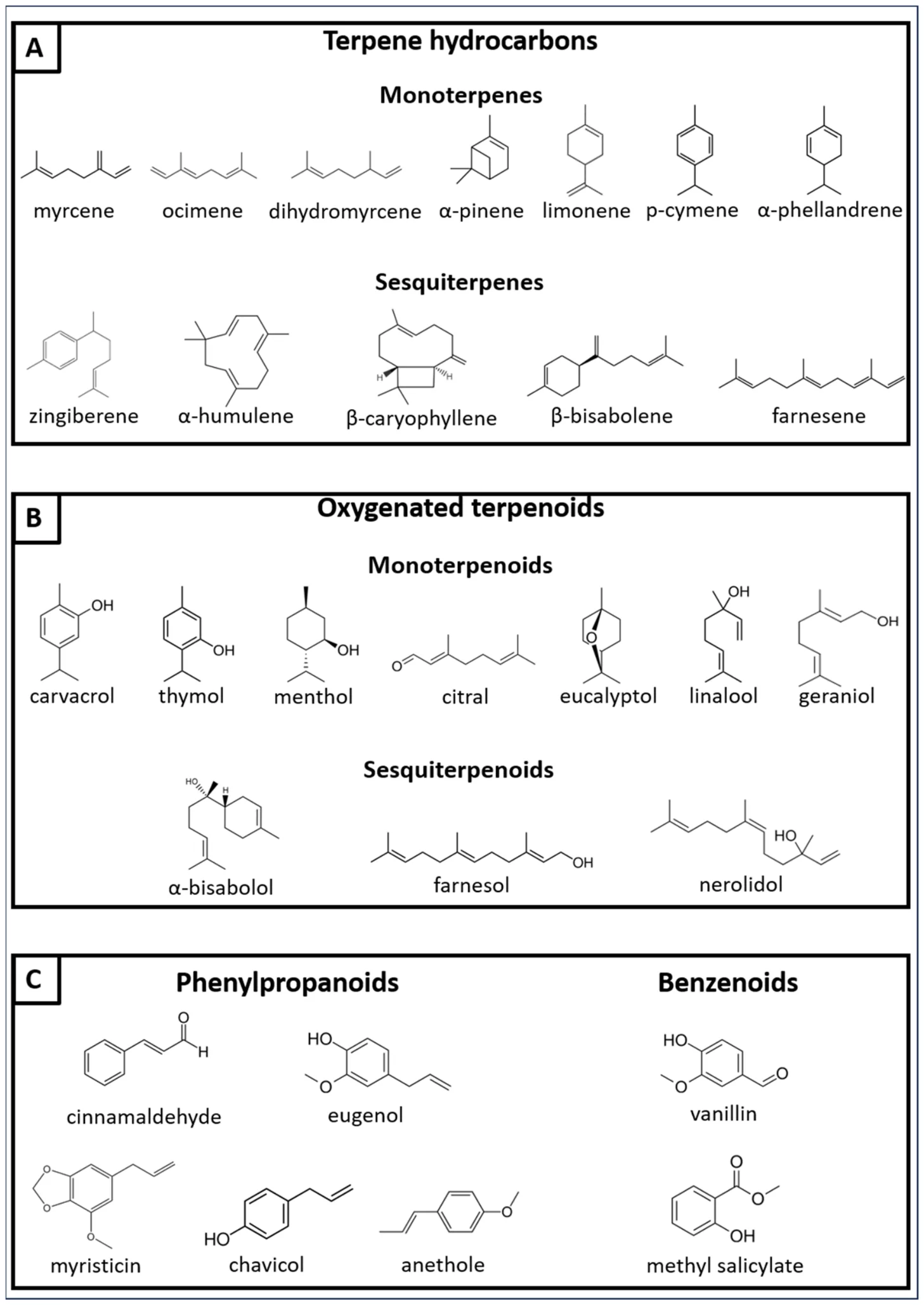
Representative chemical classes and structures of essential oil constituents. Panel (**A**) shows terpene hydrocarbons, including representative monoterpenes and sesquiterpenes. Panel (**B**) shows oxygenated terpenoids, including representative oxygenated mono- and sesquiterpenoid derivatives. Panel (**C**) shows representative phenylpropanoid and benzenoid constituents. Structures are intended as representative examples of major EO chemical classes. Abbreviation: EO, essential oil.

EOs are complex mixtures of low-molecular-weight volatile organic compounds, mainly terpenes, terpenoids and phenylpropanoid/benzenoid derivatives. These compounds are responsible for the characteristic aroma, ecological functions and many biological properties of EOs. Their relative abundance depends on the plant species, genotype, chemotype, organ, developmental stage and environmental conditions, but also on the extraction method used to recover them. Therefore, understanding the biosynthetic origin and chemical classes of EO constituents is essential for interpreting why different plant raw materials or extraction technologies may generate oils differing in yield, composition and quality [2,3,14]. Representative chemical classes of EO constituents are summarized in Figure 2.

Although EO constituents can be broadly classified into terpene hydrocarbons, oxygenated terpenoids, phenylpropanoid/benzenoid derivatives and other less common volatile compounds, the present review focuses mainly on terpene hydrocarbons, oxygenated terpenoids and phenylpropanoid/benzenoid derivatives because these classes are the most relevant for EO chemotype, volatility, extraction behavior and industrial quality. Other minor constituents, including degradation products, sulfur-containing compounds, nitrogen-containing compounds or fatty-acid-derived volatiles, may occur in specific oils or aromatic extracts, but they are generally less representative of the main volatile fraction of most EOs [31].

### 3.1. Terpenes and Terpenoids: MVA/MEP Pathways and Chemotype Formation

Terpenes and terpenoids represent the dominant chemical classes in many EOs. Terpenes are hydrocarbon compounds, whereas terpenoids include oxygenated or otherwise modified derivatives, such as alcohols, aldehydes, ketones, phenols, ethers and oxides. This distinction is important because oxygenated terpenoids often contribute strongly to fragrance quality and biological activity, whereas terpene hydrocarbons generally differ in volatility, polarity and susceptibility to losses during extraction or storage [2,14].

All plant terpenoids derive from the universal C_5_ isoprenoid precursors isopentenyl diphosphate (IPP) and dimethylallyl diphosphate (DMAPP). In plants, these precursors are produced through two compartmentally separated but metabolically connected pathways: the mevalonate pathway (MVA), associated mainly with the cytosol/peroxisome/endoplasmic-reticulum system, and the methylerythritol phosphate pathway (MEP), localized in plastids. The MEP pathway primarily supplies precursors for hemiterpenes, monoterpenes and diterpenes, whereas the MVA pathway mainly contributes to sesquiterpenes and triterpenes; however, cross-talk between the two pathways can occur and may vary according to the species, organ and developmental stage [2,15,32]. This biosynthetic organization provides the biochemical basis for the formation of terpene hydrocarbons and oxygenated terpenoids in EO-producing plants. A recent review further describes this biosynthetic organization as a multilayered regulatory network in which precursor supply, MVA/MEP pathway cross-talk, compartmentalization and regulatory mechanisms coordinate carbon flux toward different terpenoid products [33].

The sequential condensation of IPP and DMAPP generates prenyl diphosphate intermediates of different chain lengths, mainly geranyl diphosphate (GPP, C_10_), farnesyl diphosphate (FPP, C_15_) and geranylgeranyl diphosphate (GGPP, C_20_). These intermediates are the direct precursors of monoterpenes, sesquiterpenes and diterpenes, respectively. Terpene synthases convert GPP, FPP and GGPP into diverse acyclic or cyclic terpene skeletons, while downstream modifying enzymes further generate oxygenated terpenoids through reactions such as hydroxylation, dehydrogenation and acylation. Therefore, the EO chemotype is not defined only by the plant species, but also by the expression, localization and regulation of specific terpene synthases and modifying enzymes [2,14].

This biosynthetic organization has direct implications for EO composition and extraction. Monoterpenes are generally more volatile and often dominate distilled oils, whereas sesquiterpenes are less volatile and may be more affected by extraction time, temperature or solvent selectivity. Oxygenated monoterpenes and sesquiterpenes are frequently important for sensory quality and biological activity, and their relative recovery may differ among hydrodistillation, microwave-assisted extraction, solvent extraction and supercritical CO_2_ extraction. Thus, terpenoid biosynthesis provides the biochemical basis for understanding the combined effects of the genotype, environment, chemotype and extraction method on final EO composition [2,3,15].

### 3.2. Phenylpropanoids and Other Volatile Aromatic Constituents

**Figure 3:**
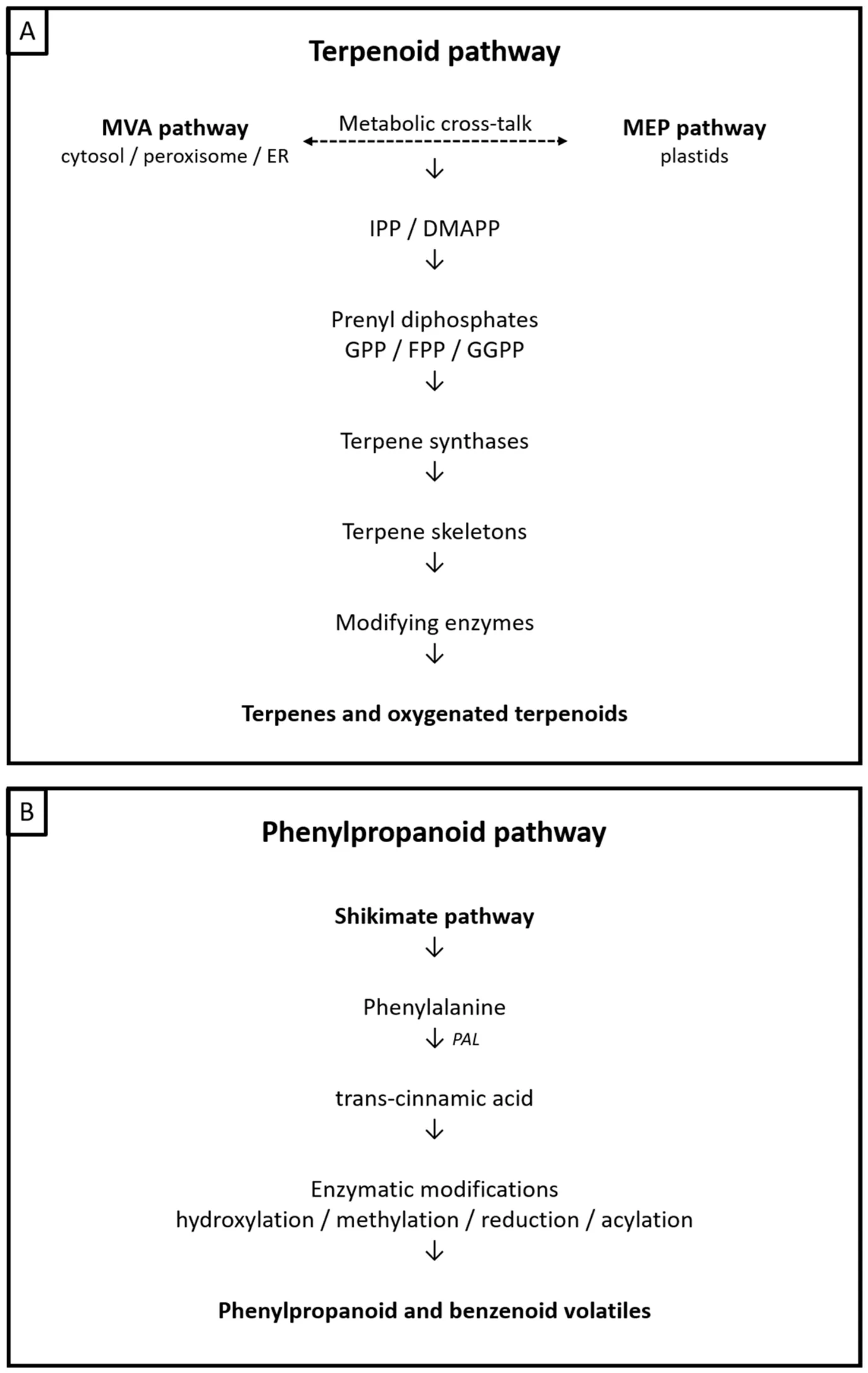
Simplified biosynthetic origin of EO constituents. (**A**) shows the terpenoid pathway, in which the universal C_5_ precursors isopentenyl diphosphate (IPP) and dimethylallyl diphosphate (DMAPP) are produced through the compartmentally separated but metabolically connected mevalonate (MVA) and methylerythritol phosphate (MEP) pathways before being converted into prenyl diphosphates, including geranyl diphosphate (GPP), farnesyl diphosphate (FPP) and geranylgeranyl diphosphate (GGPP), leading to terpene skeletons and oxygenated terpenoid derivatives. The MVA pathway is shown as mainly associated with the cytosol/peroxisome/endoplasmic-reticulum system, whereas the MEP pathway is localized in plastids. The dashed arrow indicates metabolic cross-talk between MVA- and MEP-derived precursor pools. (**B**) shows the phenylpropanoid/benzenoid pathway, originating from the shikimate/phenylalanine pathway through phenylalanine ammonia lyase (PAL) and subsequent enzymatic modifications. Abbreviations: EO, essential oil; ER, endoplasmic reticulum.

Phenylpropanoid and benzenoid derivatives constitute another important group of EO and aroma-related constituents, although their abundance varies strongly among plant species and chemotypes. They are especially relevant in oils or aromatic extracts rich in eugenol, isoeugenol, cinnamaldehyde, anethole, estragole, chavicol, methyl chavicol, myristicin or related aromatic compounds. Unlike terpenoids, phenylpropanoid and benzenoid volatiles originate mainly from the shikimate/phenylalanine pathway. Phenylalanine is converted by phenylalanine ammonia lyase (PAL) into trans-cinnamic acid, which is further transformed through hydroxylation, methylation, reduction, acylation and other enzymatic reactions into a wide variety of aromatic metabolites [2,34]. The simplified biosynthetic origin of terpenoid and phenylpropanoid/benzenoid EO constituents is summarized in Figure 3.

These compounds contribute to plant defense, UV protection, pollinator attraction, pathogen resistance and aroma formation, and they are also relevant for the sensory, antioxidant and antimicrobial properties of several EOs and aromatic extracts. From an extraction perspective, phenylpropanoids may behave differently from monoterpene hydrocarbons because they often have higher polarity, different volatility and different thermal stability. Their recovery may therefore be strongly influenced by extraction technology, especially when comparing distillation-based methods with solvent extraction, supercritical CO_2_ extraction or headspace-based analytical profiling [2,3,34].

Overall, the biosynthetic classification of EO constituents links plant metabolism with oil composition. Environmental conditions can regulate metabolic flux through terpenoid and phenylpropanoid pathways, determining which compounds are synthesized and accumulated by the plant. Extraction technology then determines which of these compounds are selectively recovered, enriched or lost in the final product and, under conditions promoting hydrolysis, oxidation, isomerization or thermal degradation, which constituents may undergo true chemical modification. This relationship between biosynthesis, chemotype and extraction selectivity provides the conceptual basis for the following section on extraction methods as determinants of EO composition, yield and sustainability [2,3,6]. To make this biosynthesis-to-extraction link more operational, Table 1 summarizes how major EO chemical classes can be translated into practical extraction risks, method-selection considerations and analytical quality-control endpoints.

## 4. Extraction Methods as Determinants of Essential Oil Composition, Yield, and Sustainability

Extraction strategy translates the biological and chemical potential of the plant matrix into a final EO or EO-related product with a defined yield and composition, application-related properties and process requirements. Therefore, the extraction strategy should be evaluated through combined criteria, including plant matrix, target chemical class, degradation risk, product boundary, final application and process sustainability [3,26].

Method-dependent variations in EO profiles should not be automatically interpreted as extraction-induced chemical transformations. Changes in relative abundance may result from selective recovery or partitioning, incomplete release from plant tissues, volatile losses, water-dependent solubility or method-dependent analytical artifacts. True transformation should be reserved for chemical conversion processes, including hydrolysis, oxidation, isomerization or heat-induced degradation/rearrangement of thermolabile constituents. For instance, during lavender oil extraction, water-contact conditions have been reported to promote linalyl acetate degradation/hydrolysis to linalool, whereas broader thermal degradation pathways of aroma and EO constituents have been reviewed elsewhere [35,36].

Different technologies applied to the same plant material can produce products that are not chemically, sensorially or functionally equivalent because they differ in selectivity, degradation risk and process requirements. Their comparison should therefore consider composition, extraction efficiency, resource use, scalability and final application, although heterogeneous matrices, operating parameters and reporting formats limit direct cross-study evaluation [3,26,27,28].

Accordingly, sustainability-related advantages discussed in this review should be interpreted as process- and context-dependent. When quantitative indicators such as energy consumption, water or solvent use, CO_2_ emissions, solvent recovery or yield-normalized performance are not reported in the original studies, sustainability is considered as an inferred process-related advantage rather than as a demonstrated environmental benefit. Similarly, yield, energy-use and bioactivity comparisons are interpreted primarily within individual studies, because fresh- or dry-mass yield bases, batch size, energy denominators and assay conditions are often not harmonized across the literature.

Total phenolic content (TPC) and total flavonoid content (TFC) values determined using colorimetric assays in EO studies should be interpreted as assay-dependent functional indicators rather than as direct evidence of EO composition, because Folin–Ciocalteu and aluminum chloride assays may be influenced by sample composition, compound structure and non-target reactions [37,38]. Therefore, GC–FID and GC–MS profiles remain the primary evidence for EO volatile composition.

Parameters such as plant organ, particle size, moisture content, solvent-to-solid ratio, temperature, extraction time, pressure, microwave or ultrasound power and post-extraction separation can all influence the final EO profile. For this reason, the following sections describe the main extraction techniques by considering not only their operating principles, but also their impact on EO composition, degradation risk, recovery of target compounds, resource consumption, scalability and suitability for sustainable production [3,26,27]. A comparative overview of the essential-oil-related approaches discussed below is provided in Table 2, distinguishing classical EO recovery methods, EO-related extractive approaches, pretreatment/intensification strategies and analytical volatile-profiling techniques. Detailed operating variables, selectivity, degradation risks and scale-up constraints are discussed in the corresponding subsections.

### 4.1. Steam Distillation and Its Impact on Essential Oil Yield and Composition

Steam distillation (SD) is one of the most widely used conventional methods for EO extraction and remains an industrial benchmark because of its simplicity, scalability, absence of organic solvents and relatively low equipment cost. In SD, steam passes through the plant matrix without immersing the material directly in boiling water; volatile compounds are entrained by the steam, condensed and then separated from the aqueous phase according to density differences. This operational distinction from hydrodistillation is relevant because, in hydrodistillation, the plant material is placed in boiling water, whereas in steam distillation only steam passes through the plant matrix [3,27].

In SD, extraction time, steam flow, plant particle size, moisture content, packing density and plant organ can influence mass transfer, volatile recovery and the final EO profile. Therefore, SD conditions should be optimized according to plant matrix, target chemical classes, desired chemotype and intended application rather than applied as a generic extraction method [3].

Comparative studies demonstrate that SD can produce oils with different yield, composition and functional properties compared with other distillation-based methods applied to the same plant material. In *Ferulago contracta*, *Salvia rosmarinus* and *Lavandula sublepidota*, SD produced a higher EO yield, total phenolic content, total flavonoid content and antioxidant activity than hydrodistillation. GC–MS analysis also showed method-dependent differences in the relative abundance of major volatile constituents, confirming that the distillation method can substantially affect EO composition and antioxidant capability [26].

A method-dependent effect was also observed for *Lavandula* × *intermedia* ‘Margaret Roberts’, where SD, hydrodistillation and cellulase-assisted hydrodistillation differed in extraction time, energy consumption, camphor content and sensory properties. SD required a longer average extraction time than hydrodistillation and cellulase-assisted hydrodistillation, 57 min versus 51 and 49 min, respectively, and slightly higher energy consumption than hydrodistillation, 15.0 versus 13.4 kJ/g oil. However, SD provided more consistent analytical results, produced oils with the highest camphor content and generated a stronger, more plant-like odor, whereas cellulase-assisted hydrodistillation produced oils with lower camphor content and a sweeter, more pleasant odor [27].

The limitations of SD are mainly related to heat, moisture and process duration. Prolonged exposure to hot steam may promote the loss of highly volatile compounds or changes in thermolabile constituents, and the final oil composition may vary according to the matrix and target compounds. Where robustness, low cost, solvent-free processing and scale-up are priorities, SD remains a useful benchmark; however, its performance should be assessed against alternative or intensified technologies using measurable criteria such as EO yield, target compound preservation, extraction time, energy consumption, biological activity and environmental impact [3,26,27].

Modified steam-based technologies may partly overcome some limitations of conventional SD. In *Syzygium aromaticum* L., superheated steam distillation was compared with hydrodistillation and conventional SD. The extraction method significantly affected EO yield, chemical composition, antioxidant activity and antimicrobial activity; superheated steam distillation produced the highest EO yield, whereas conventional SD produced the lowest yield. Superheated steam-distilled EO also showed stronger antioxidant and antimicrobial activities, while GC–MS confirmed eugenol and caryophyllene as major constituents of the oils [57]. These findings indicate that steam-based extraction should not be considered a single fixed approach, but rather a family of processes whose operating conditions can strongly influence EO yield, composition and sustainability.

### 4.2. Hydrodistillation and Its Impact on Essential Oil Yield and Composition

Hydrodistillation (HD) is one of the most established conventional methods for essential oil (EO) extraction and is widely used as a reference technique in laboratory-scale studies and pharmacopeial procedures. In HD, the plant material is immersed in water and heated to boiling; volatile constituents are released from the matrix, co-distilled with water vapor, condensed and separated from the hydrolate according to density differences. This method is simple, inexpensive, solvent-free and broadly applicable to different aromatic matrices, but the direct contact between plant material, boiling water and prolonged heating makes HD particularly sensitive to process parameters such as extraction time, water-to-plant ratio, particle size, soaking time and matrix structure [3].

Hydrodistillation should therefore be considered not only as a recovery method, but also as a factor capable of modifying EO yield and composition. Distillation time is especially important. In lemon thyme (*Thymus* × *citriodorus*), HD performed at different distillation times showed that EO yield did not increase uniformly throughout the process; after 60 min, the increase in oil percentage became minimal, whereas longer distillation decreased the relative percentage of monoterpenes and increased the percentage of sesquiterpenes. The authors concluded that HD longer than 60 min was not useful for this matrix, indicating that excessive distillation may increase energy consumption without proportionally improving oil recovery and may also shift the EO profile [58].

Other operational variables can also influence HD efficiency. In patchouli (*Pogostemon cablin*) EO extraction, response surface methodology was used to optimize HD parameters, including extraction time, solid–liquid ratio, soaking time and particle size. The optimized conditions were an extraction time of 130 min, a solid–liquid ratio of 1:18 g:mL, a soaking time of 88 min and a particle size of 0.2 mm, resulting in an actual EO yield of 1.21%. This study showed that HD efficiency depends on the combined optimization of heat and mass transfer conditions rather than on extraction time alone [59].

HD performance depends strongly on the plant matrix, target constituents and intended application. In citron (*Citrus medica* L.) peel, both HD and supercritical CO_2_ extraction were used to valorize a fruit-processing by-product. Supercritical CO_2_ extraction produced a higher yield, higher total phenolic and flavonoid contents, and a higher limonene percentage than HD, supporting its potential as a greener and more selective technique. Nevertheless, the study also confirms that HD remains a relevant conventional benchmark for evaluating alternative extraction processes, especially in comparative studies focused on yield, volatile composition and bioactivity [39].

### 4.3. Microwave-Assisted Hydrodistillation and Solvent-Free Microwave Extraction

Microwave-assisted hydrodistillation (MAHD) is an intensified extraction technique that combines conventional hydrodistillation with microwave heating. Microwaves are non-ionizing electromagnetic waves, commonly used at 2450 MHz in laboratory and domestic microwave systems, and their heating effect is mainly related to the interaction of the electric field with polar molecules and ionic species. The two main mechanisms responsible for microwave heating are dipole rotation and ionic conduction, which convert electromagnetic energy into heat within the irradiated matrix [60,61].

In EO extraction, this heating mode is relevant because microwave energy can be absorbed by water naturally present in plant tissues or by water added during hydrodistillation. The resulting internal heating promotes evaporation, pressure build-up and weakening or rupture of oil glands and cell walls, thereby accelerating the release of volatile constituents. Unlike conventional hydrodistillation, where heat is transferred mainly from the outside to the inside of the matrix by conduction and convection, microwave-assisted extraction can generate volumetric heating and align heat and mass transfer more efficiently, which may reduce extraction time and energy losses [3,61]. A recent review focusing specifically on microwave-assisted extraction of essential oils further emphasizes that microwave power, extraction time, solvent-to-raw-material ratio, system configuration and process control are key variables influencing EO yield, quality and technological feasibility [42].

Experimental comparisons confirm that MAHD can improve process efficiency while maintaining, or slightly modifying, EO composition. In *Salvia rosmarinus*, MAHD and conventional Clevenger hydrodistillation produced comparable EO yields, but MAHD reduced the extraction time from 180 to 20 min for the laboratory-scale extraction of 100 g of dried rosemary. Under these experimental conditions, the reported electric consumption decreased from 2.25 to 0.23 kWh, and the estimated CO_2_ emissions decreased from 1800 to 184 g. The oils obtained by the two methods were qualitatively similar, but MAHD showed a slightly higher percentage of total oxygenated compounds and a lower percentage of non-oxygenated compounds than Clevenger hydrodistillation [40].

A similar improvement in process efficiency was observed in coriander seeds. Hydrodistillation and MAHD produced similar EO yields, 0.31% and 0.325%, respectively, but MAHD required a shorter extraction time, 60 min compared with 240 min for hydrodistillation, and lower energy consumption, 0.63 kWh compared with 2.52 kWh. MAHD-derived coriander EO also showed higher total phenolic content, stronger 2,2-diphenyl-1-picrylhydrazyl (DPPH) radical-scavenging activity and slightly higher oxidative stability than hydrodistilled oil, while GC–MS analysis identified linalool as the main constituent in both oils [41].

Microwave-based approaches may also affect the relative abundance of chemical classes and biological properties. In three *Curcuma* species, conventional hydrodistillation was compared with solvent-free microwave extraction (SFME). Although the major EO constituents obtained by the two methods were qualitatively similar, their quantitative profiles differed substantially: hydrodistilled oils were dominated by hydrocarbon compounds, whereas SFME-derived oils were richer in oxygenated compounds. SFME also required less extraction time, energy and water, produced less waste, gave higher EO yields and generated oils with stronger antioxidant, anti-tyrosinase and anticancer activities than hydrodistillation [28].

A comparative study on *Rosa* × *damascena* fresh petals further showed that extraction strategy can markedly affect EO profiles, with conventional EOs obtained by SD and HD dominated by oxygenated terpenes and microwave-mediated EOs obtained by microwave hydrodiffusion and gravity (MHG) and SFME dominated by alcohols [62].

Taken together, these results show that microwave-assisted methods affect more than extraction time. By changing heat transfer, extraction kinetics and plant-cell disruption, they can influence EO yield, oxygenated/hydrocarbon ratios, phenolic-associated antioxidant properties and other biological activities. From a green extraction perspective, MAHD and SFME are consistent with the principles of process intensification, energy saving, reduced solvent or water use and production of high-quality EOs, provided that the process is properly optimized [3,63].

However, MAHD and SFME are not automatically superior for all plant matrices. Their performance depends on plant moisture, particle size, water-to-plant ratio, microwave power, irradiation time, sample loading, reactor geometry and condensation efficiency. Excessive microwave power or prolonged irradiation may alter EO profiles or promote degradation of thermolabile constituents. Moreover, scale-up remains a critical issue because microwave penetration depth, non-uniform heating and equipment cost may affect reproducibility and industrial feasibility. Therefore, MAHD and SFME should be evaluated using integrated criteria, including EO yield, extraction time, energy and water consumption, CO_2_ emissions, preservation of target oxygenated compounds, biological activity, safety and scalability.

### 4.4. Supercritical CO_2_ Extraction in EO-Related Production: Volatile-Rich CO_2_ Extracts and Selectivity

Supercritical fluid extraction (SFE), particularly supercritical CO_2_ extraction, is an advanced extraction technology used in EO-related production to recover plant volatiles, volatile-rich CO_2_ extracts, flavors, fragrances and other lipophilic bioactive compounds from vegetable matrices. Carbon dioxide becomes supercritical above its critical temperature and pressure, approximately 31 °C and 7.38 MPa, acquiring gas-like diffusivity and liquid-like solvating power. These properties allow supercritical CO_2_ to penetrate plant tissues efficiently and dissolve non-polar or moderately non-polar compounds, while being easily removed from the resulting CO_2_ extract by depressurization. Because CO_2_ is non-flammable, non-toxic, chemically inert, relatively inexpensive and recyclable, SFE is considered a green alternative to conventional solvent extraction and, in selected cases, to distillation-based EO extraction [3,43,44].

The main advantage of supercritical CO_2_ extraction is not only the replacement of organic solvents, but also its tunable selectivity. By modifying pressure and temperature, the density and solvent strength of CO_2_ can be adjusted, allowing selective extraction of different volatile, semi-volatile or lipophilic fractions. This is particularly relevant for EO production because SFE may reduce the thermal and hydrolytic degradation associated with long hydrodistillation or steam distillation processes. However, the product obtained by SFE may not be identical to a distilled EO: depending on operating conditions, supercritical CO_2_ can also co-extract waxes, pigments, fatty acids, sterols, tocopherols, phenolics or other less volatile lipophilic compounds, producing a chemically enriched CO_2_ extract or an oleoresin-like fraction rather than a classical EO [43,44,64].

For this reason, SFE should be evaluated according to the target product. If the aim is to obtain a fragrance-grade EO, extraction conditions should favor volatile terpenes and oxygenated terpenoids while limiting the recovery of heavier non-volatile fractions. If the aim is a functional CO_2_ extract for food, cosmetic, nutraceutical or pharmaceutical applications, the co-extraction of lipophilic antioxidants, phenolics, tocopherols, phytosterols or fatty acids may instead be advantageous. Therefore, in SFE, extraction performance cannot be defined only by total yield, but by the selective recovery of a chemical profile suitable for the intended application [43,44,64].

Several parameters strongly influence SFE selectivity and composition. Pressure is one of the most important variables because it controls CO_2_ density and solvating power; increasing pressure may enhance the recovery of heavier or less volatile compounds. Temperature has a dual effect because it can increase solute vapor pressure while decreasing CO_2_ density at constant pressure. Other relevant factors include CO_2_ flow rate, extraction time, particle size, moisture content, matrix structure and the use of co-solvents or modifiers when more polar compounds are targeted. Consequently, SFE optimization must be matrix-specific, because the same pressure–temperature conditions may produce different results depending on plant organ, moisture, particle size and target chemical class [3,44,64].

Comparative studies show that supercritical CO_2_ extraction can produce CO_2_ extracts that differ substantially from EOs obtained by hydrodistillation. In citron (*Citrus medica* L.) peel, a fruit-processing by-product, supercritical CO_2_ extraction produced higher extraction yield than hydrodistillation, 7.6% versus 4.5%, under milder thermal conditions, 35 °C and 100 bar for 30 min compared with 100 °C for 180 min in hydrodistillation. The supercritical CO_2_ sample also showed a higher limonene percentage by GC–MS and stronger antioxidant activity than the hydrodistilled EO. The same study reported higher colorimetric TPC and TFC values for the SFE-CO_2_ sample, but these values are treated here as assay-dependent functional indicators rather than direct compositional evidence of EO volatile chemistry [39].

In *Dalbergia odorifera* heartwood, hydrodistillation and supercritical CO_2_ extraction generated products with different yields, compositions and antimicrobial activities. Supercritical CO_2_ extraction produced a higher yield and a broader range of detected constituents than hydrodistillation, whereas the antimicrobial profile differed according to the tested microorganism. This comparison shows that SFE can alter yield, composition and biological activity, with method suitability depending on the target compounds and biological endpoint [45].

Despite its advantages, SFE has important limitations. The equipment is expensive, the process requires high-pressure operation, and optimization can be technically demanding. Supercritical CO_2_ is intrinsically more suitable for non-polar and moderately non-polar compounds; extraction of highly polar constituents may require co-solvents, which partially modifies the solvent-free character of the process. Moreover, industrial implementation requires careful control of pressure, temperature, CO_2_ flow, solvent recovery, extractor packing, process safety and scale-up. A techno-economic and safety assessment of a closed-cycle supercritical CO_2_ process for essential oils and plant extracts showed that solvent recovery and recycling can reduce waste and emissions, but the process requires capital investment, high-pressure equipment and regular control of pressure, temperature and flow deviations [46].

Overall, supercritical CO_2_ extraction is particularly valuable when the objective is to obtain organic-solvent-free, volatile-rich or chemically enriched CO_2_ extracts with minimal solvent residues under mild thermal conditions. Its potential contribution to more sustainable EO-related production lies in its selectivity, reduced use of organic solvents, recyclability of CO_2_ and potential for valorizing plant by-products, provided that energy demand, CO_2_ recovery, equipment requirements and process scale are also considered. However, its suitability should be assessed using integrated criteria, including EO yield or CO_2_ extract yield, EO profile or volatile profile according to product boundary, co-extracted non-volatile fraction, biological activity, energy demand, equipment cost, CO_2_ recycling, safety, scalability and final product application.

### 4.5. Solvent Extraction: Concretes, Absolutes and Solvent-Related Quality Constraints

Solvent extraction is a conventional technique used to recover aromatic and lipophilic compounds from plant matrices by dissolving them in an organic solvent. In the context of EO-related products, this method should be clearly distinguished from hydrodistillation and steam distillation because it often does not produce a classical distilled EO, but rather a concrete, absolute, resinoid, oleoresin or volatile-rich aromatic extract containing both volatile and non-volatile constituents. This distinction is important for product classification, quality control and final application, especially in the fragrance industry, where plant extracts and absolutes are valued for their rich and persistent aroma profiles [48,65].

Solvent extraction is particularly relevant for delicate floral materials, resins and matrices whose aromatic profile may be poorly represented or partially altered by distillation. In oil-bearing rose, for example, the main industrial products include rose oil, rose hydrolate, rose concrete and rose absolute. Rose concrete and rose absolute are obtained by solvent extraction. In this process, fresh flowers are extracted with a non-polar solvent, such as n-hexane, which dissolves aromatic compounds together with waxes, pigments and other soluble substances; after solvent removal under vacuum, the waxy mass is known as concrete. The concrete is then treated with ethanol at low temperature to dissolve the aromatic constituents while leaving behind waxes and paraffinic substances, producing the rose absolute after alcohol evaporation [65].

The composition of solvent-derived aromatic extracts depends strongly on solvent polarity, extraction time, temperature, solvent-to-solid ratio, plant particle size and solvent-removal conditions. Organic solvent extraction can give higher yields than hydrodistillation in some aromatic plants, but the resulting product is chemically broader and may contain non-volatile fractions; therefore, higher yield should not automatically be interpreted as higher product quality. Zhou et al. report that organic solvent extraction may give higher yield and different composition compared with hydrodistillation, but also that organic reagents are difficult to completely separate from solutes and fully recycle, and that solvent removal with a rotary evaporator may cause loss of volatile constituents during evaporation [3].

Solvent choice is therefore a key determinant of the quality of the resulting aromatic extract. In *Rosa* × *damascena*, multistage extraction with ethyl acetate, ethanol, hexane and petroleum ether produced concretes with different GC–MS profiles. Ethyl acetate extracted the highest number of detected components, whereas hexane and petroleum ether produced more similar constituent profiles. Phenyl ethyl alcohol and 5-hydroxymethylfurfural were detected in all four solvent-derived concretes, and the study concluded that solvent characteristics significantly influenced the composition of *Rosa* × *damascena* concrete [47].

The comparison among headspace analysis, distillation and solvent extraction further confirms that the extraction approach determines the aromatic product obtained. In *Rosa* × *damascena*, headspace solid-phase microextraction (HS-SPME) identified a broader volatile profile in fresh flowers than hydrodistilled rose EO, which was dominated by monoterpene alcohols such as geraniol, citronellol and nerol. By contrast, phenylethyl alcohol was prominent in fresh flowers, rose hydrolate and residue water, showing that water solubility, distillation and extraction processes redistribute rose scent compounds among the different products. In the same study, different non-polar solvents also produced different concrete and absolute yields, demonstrating that solvent extraction is not a neutral procedure but a determinant of both yield and scent composition [65].

The main limitations of solvent extraction are related to safety, environmental impact and product purity. Traditional organic solvent extraction may involve toxic or non-renewable solvents, high solvent consumption, long processing times, solvent recovery steps and possible residual solvent traces in the final product. These aspects are particularly relevant for food, cosmetic and other regulated applications, where residual solvents and quality requirements must be carefully controlled. In green extraction terms, solvent extraction should therefore be evaluated not only by yield and aroma recovery, but also by solvent toxicity, recyclability, energy use, waste production, worker safety and purity of the resulting aromatic extract [48,63].

Accordingly, solvent extraction remains useful for selected high-value aromatic matrices, especially when the target is a concrete or absolute rather than a distilled EO. However, from a sustainable production perspective, it should be optimized and compared with greener alternatives, including ethanol-based extraction, bio-based solvents, supercritical CO_2_ extraction, ultrasound-assisted extraction, microwave-assisted extraction and solvent-free approaches. The suitability of solvent extraction should be assessed using integrated criteria: target aroma recovery, volatile/non-volatile balance, solvent selectivity, residual solvent level, extraction yield, sensory quality, regulatory acceptability and environmental footprint.

### 4.6. Ultrasound-Assisted Extraction as a Pretreatment in EO Recovery

Ultrasound-assisted extraction (UAE) is an intensification strategy used to improve the recovery of EOs, aromatic extracts and other plant bioactive compounds. Ultrasound consists of mechanical waves that propagate through a liquid medium and generate alternating compression and rarefaction cycles. The main phenomenon responsible for extraction enhancement is acoustic cavitation, namely the formation, growth and collapse of microbubbles in the extraction medium. Bubble collapse can generate localized shear forces, microjets, turbulence and physical disruption of plant tissues, thereby improving solvent penetration, cell-wall permeability and mass transfer of intracellular or glandular compounds [3,63].

The extraction-enhancing effect of ultrasound is not based on a single mechanism. Chemat et al. described several physical effects induced by ultrasound in plant matrices, including fragmentation, erosion, sonocapillary effect, sonoporation, local shear stress and detexturation. These effects can increase the contact surface between plant material and solvent, facilitate solvent penetration into plant tissues and accelerate the release of target molecules. Importantly, UAE performance depends strongly on plant matrix, pretreatment, particle size, drying conditions and accessibility of the target compounds; therefore, ultrasound should be optimized according to the raw material rather than applied as a generic treatment [63].

In EO production, UAE is frequently used as a pretreatment before hydrodistillation or in combination with other techniques, rather than as a stand-alone method for obtaining classical distilled EOs. In citrus waste by-products, ultrasound-assisted pretreatment before hydrodistillation increased EO recovery by approximately 33%, reduced extraction time from 120 to 80 min and was economically more favorable than conventional hydrodistillation. The same study evaluated EO functional properties by Fourier-transform infrared spectroscopy (FTIR) and GC–MS and also showed that ultrasound improved the extraction of antioxidant compounds from citrus peel and pulp/pomace, supporting the use of UAE for by-product valorization [50].

A similar effect was observed in Tribute citrus peel. Ultrasound-assisted pretreatment produced an EO yield approximately 33% higher than hydrodistillation while requiring a shorter extraction time. GC–MS analysis showed that terpenes were the predominant compounds in both oils and that d-limonene remained the major constituent, accounting for more than 86% of the total volatile concentration in both hydrodistillation and ultrasound-assisted pretreatment extraction. Electronic-nose analysis also indicated no significant difference between the volatile aroma profiles obtained by the two methods, suggesting that ultrasound pretreatment increased yield without markedly altering the dominant citrus aroma profile [51].

Ultrasound can also be integrated with green solvent strategies to produce aromatic extracts that are not classical distilled EOs. In *Thymus vulgaris*, ultrasound extraction using sunflower oil as a natural solvent was proposed for the production of a green absolute. The optimized conditions were 50 °C, 22 min and 98 W, and ultrasound extraction increased absolute yield by 47% compared with conventional sunflower-oil extraction. The ultrasound-derived absolute showed high recovery of the monoterpene phenols thymol and carvacrol, was free from waxes and organic solvent residues, and showed stronger antioxidant activity than the conventionally produced absolute. This study shows that UAE can improve extraction kinetics, facilitate solvent substitution and enhance product sustainability, although the final product is an absolute rather than a classical EO [52].

UAE performance is strongly condition-dependent. Its outcome depends on ultrasound frequency, amplitude, real acoustic power delivered to the medium, sonication time, temperature, solvent properties, solvent-to-solid ratio, particle size, reactor geometry and sample loading. Excessive sonication or poorly controlled temperature may alter sensitive constituents, while inefficient acoustic-field distribution may reduce reproducibility. Vinatoru emphasized that many UAE studies are difficult to reproduce because they do not adequately report the physical state of the plant material, particle size, residual moisture, ultrasonic frequency, real acoustic power, vessel geometry or actual extraction temperature. The same author also warned that laboratory-scale UAE is not linearly scalable to industrial volumes because acoustic-field distribution and cavitation behavior change substantially with reactor size [53].

UAE can reduce extraction time, solvent use and energy demand, particularly when used as a pretreatment for hydrodistillation or combined with green solvents. However, these advantages should be demonstrated using measurable indicators and evaluated alongside product yield and composition, reproducibility, scalability and waste generation.

### 4.7. Headspace Techniques for Volatile Profiling and Essential Oil Quality Control

Headspace techniques are solvent-free analytical sampling approaches used to characterize volatile organic compounds released from solid or liquid plant matrices, essential oils, hydrolates, concretes, absolutes or other aromatic products. In a sealed vial, volatile compounds partition between the sample phase and the gas phase above it, known as the headspace. After a defined equilibration time and temperature, the volatile fraction present in the headspace can be sampled directly or concentrated using sorbent- or microextraction-based approaches and then analyzed mainly by gas chromatography coupled to mass spectrometry. Unlike hydrodistillation, steam distillation, solvent extraction or supercritical CO_2_ extraction, headspace techniques are generally not productive extraction methods for obtaining commercial essential oils, but analytical tools for volatile profiling, quality control, rapid screening and comparison among extraction-derived products [3,56].

This distinction is important because the volatile profile detected by headspace analysis may differ substantially from the composition of a distilled EO or a solvent-derived absolute. Headspace analysis reflects the compounds released into the gas phase under defined analytical conditions, whereas distillation and solvent extraction depend on heat, water, solvent polarity, diffusion and matrix disruption. Therefore, headspace techniques can help identify which volatile constituents are naturally emitted by the plant material and which compounds are preferentially recovered, lost, redistributed or transformed during extraction. In *Rosa* × *damascena*, HS-SPME, hydrodistillation and solvent extraction produced markedly different scent profiles. Fresh flowers analyzed by HS-SPME showed a broader floral volatile profile and were characterized by compounds such as phenylethyl alcohol, citronellol and geraniol, whereas hydrodistilled rose EO was dominated by monoterpene alcohols such as geraniol, citronellol and nerol. Phenylethyl alcohol, abundant in fresh flowers and rose hydrolate, was present at low levels in hydrodistilled rose EO, confirming that extraction and distillation processes redistribute rose scent compounds among rose oil, rose hydrolate, concrete and absolute [65].

A similar difference between headspace analysis and hydrodistillation was reported for *Lagoecia cuminoides*. Hydrodistillation produced an essential oil richer in oxygenated monoterpenes and dominated by thymol, whereas headspace analysis mainly detected monoterpene hydrocarbons, especially γ-terpinene and p-cymene, with a lower relative abundance of thymol. This example shows that headspace volatile profiles and EO profiles are not interchangeable: the former reflects the volatile fraction released under analytical conditions, while the latter reflects the compounds recovered after heating, water contact and condensation. Headspace analysis also required less sample material and shorter analytical time than hydrodistillation, supporting its usefulness as a rapid volatile-profiling technique rather than as a productive EO extraction method [54].

Among headspace approaches, static headspace analysis allows volatile compounds to equilibrate between the sample and gas phase before direct sampling. Dynamic headspace techniques improve sensitivity by continuously purging volatile compounds from the sample with an inert gas and trapping them on a sorbent. HS-SPME is widely used because a coated fiber is exposed to the headspace, where it selectively adsorbs or absorbs volatile compounds before direct thermal desorption in the injector of a gas chromatograph (GC). Zhou et al. describe solid-phase microextraction (SPME) as a solvent-free sample pretreatment and enrichment technology integrating sampling, extraction, concentration and injection, with direct extraction, headspace SPME and membrane-protected SPME as basic modes [3].

The analytical profile obtained by HS-SPME depends strongly on experimental conditions. In Chinese chive (*Allium tuberosum*), Xie et al. optimized several HS-SPME parameters, including fiber type, sample weight, salt addition, extraction temperature, equilibration time, extraction time and desorption time. The optimized method allowed the identification of a broad volatile profile, showing that HS-SPME results are method-dependent analytical fingerprints. Changes in fiber coating, salt addition, temperature or extraction time can alter the number and relative abundance of detected volatiles; therefore, HS-SPME conditions must be carefully standardized when the method is used for chemotype comparison, quality control or authenticity studies [55].

Headspace-based microextraction can also be useful for rapid screening and bioprospecting of essential-oil-bearing plants. Letseka et al. developed a headspace bubble-in-drop single-drop microextraction method requiring only small amounts of plant material and short sampling times. The method was applied to *Pinus radiata*, *Tagetes minuta* and *Artemisia afra*, detecting VOCs previously reported in essential oils from these or related plants. The authors proposed the approach as a rapid, low-sample and low-cost screening tool for EO-bearing plants, particularly useful when only limited plant material is available or when many samples must be compared [56].

Overall, headspace techniques function as analytical and quality-control tools that support sustainable EO production rather than as industrial extraction methods. They can help identify native aroma markers, compare fresh plant material with extracted EOs or absolutes, distinguish native volatile emissions from extraction-derived profiles by highlighting volatile losses, redistribution among product fractions, selective enrichment or true extraction-related chemical modifications when supported by compositional evidence, monitor storage-related changes and support chemotype or authenticity studies. However, they do not provide total EO yield and do not necessarily represent the complete chemical composition of the plant matrix or commercial EO; they represent the volatile fraction released under selected analytical conditions. For this reason, headspace analysis should be integrated with GC–MS characterization, chemometric analysis and sensory evaluation when the objective is to link plant raw material, extraction method, volatile profile and final EO quality.

### 4.8. Standardization, Authenticity and Analytical Quality Control

Essential oil quality should be verified through botanical authentication, compositional profiling and appropriate quality-control specifications, rather than by yield, aroma or biological activity alone. Practical quality assurance should consider traceable plant identity, plant organ, chemotype when relevant, geographical origin, harvest and post-harvest conditions and, when possible, voucher material or equivalent traceability information. Analytical verification relies primarily on gas chromatography–flame ionization detection (GC–FID) and gas chromatography–mass spectrometry (GC–MS), supported by retention indices and reference standards for major or marker constituents [66,67]. Where relevant, enantiomeric analysis, physicochemical parameters such as density, refractive index and optical rotation, oxidation or degradation markers, adulteration testing and pharmacopeial or International Organization for Standardization (ISO) specifications can provide additional evidence of authenticity, stability and conformity [66]. Chromatographic profiles can also be used in EO standardization because ISO 11024-1 describes their preparation for presentation in standards and defines representative and characteristic components with acceptable concentration limits [67]. For EO-related aromatic extracts and CO_2_ extracts, quality control should also consider product boundary, residual solvents where applicable, co-extracted non-volatile fractions and intended application.

To summarize the practical implications of the extraction strategies discussed above, selected representative comparative studies on EO-related outcomes are reported in Table 3.

Overall, the comparative evidence supports a decision-rule approach rather than a universal ranking of extraction methods. For classical EO recovery, SD, HD and microwave-assisted distillation remain the most relevant options, with method choice depending on matrix structure, heat sensitivity and process efficiency. When the objective is a volatile-rich or broader lipophilic fraction, SFE-CO_2_ or solvent extraction may be more appropriate, but the resulting product should be interpreted according to its product boundary. Headspace-based methods are best considered analytical tools, whereas ultrasound is more appropriately positioned as a pretreatment or intensification strategy. The main evidence gaps concern the limited harmonization of yield denominators, energy metrics, biological assays and product definitions across studies, providing the basis for the limitations and future research priorities outlined in the following subsection.

### 4.9. Limitations and Future Research Priorities

As reflected in the comparative studies discussed in Section 4 and summarized in Table 3, the evidence base on EO extraction remains heterogeneous, which limits direct comparison among studies. Botanical materials often differ in species, cultivar, chemotype, plant organ, developmental stage, moisture state, particle size and post-harvest handling. Extraction studies also vary in yield denominators, batch size, extraction scale, operating conditions and reporting format. Energy consumption, water demand, solvent recovery, CO_2_ recycling and waste generation are not always quantified, while GC-based compositional data may differ according to sample preparation, quantification approach, retention-index reporting and use of reference standards. Biological assays are also variable in terms of test systems, concentrations, endpoints and exposure conditions, limiting direct comparison of antioxidant, antimicrobial or cytotoxic effects across studies.

Future research should prioritize harmonized reporting of plant material, extraction conditions and analytical methods. Authenticated botanical material, chemotype documentation, clear fresh- or dry-mass yield bases, quantitative mass and energy balances, standardized GC–FID/GC–MS quality control and transparent reporting of biological assay conditions would improve comparability. More pilot- and industrial-scale studies are also needed to evaluate process reproducibility, safety, cost, solvent or CO_2_ recovery, by-product valorization and life-cycle performance. One recent case study combined experimental data with process simulation and life-cycle assessment (LCA) to generate an industrial-scale life-cycle inventory for EO extraction from *Eucalyptus intertexta*, using a defined functional unit and explicit system boundaries while accounting for co-product allocation and waste valorization [68]. This framework illustrates how scale-up and environmental performance can be evaluated when direct industrial inventory data are limited. These priorities would support the transition from method comparison based mainly on yield or selected markers toward application-oriented, scalable and environmentally documented EO production.

## 5. Conclusions

In conclusion, essential oil quality results from the interaction between plant biosynthesis, environmental regulation and extraction technology. Medicinal and aromatic plants synthesize volatile compounds mainly through terpenoid and phenylpropanoid pathways, but the final EO profile is shaped by the species, chemotype, plant organ, developmental stage, cultivation conditions and environmental stress. These factors influence biomass production, EO yield and the relative abundance of key volatile constituents, thereby affecting sensory properties, compositional reproducibility, product standardization, safety, stability and application-related bioactivity. Accordingly, biological activities such as antioxidant, antimicrobial or anticancer effects should be interpreted as application-related endpoints, not as universal markers of EO quality or substitutes for compositional standardization or authenticity assessment.

Across the reviewed evidence, extraction acts as a selection and stabilization step: it can selectively recover, enrich, preserve or lose specific compounds and, in some cases, promote true chemical modification of sensitive constituents. Conventional methods such as hydrodistillation and steam distillation remain valuable because of their simplicity, low cost and scalability, whereas intensified and complementary approaches such as microwave-assisted methods, ultrasound pretreatment and supercritical CO_2_ extraction may improve extraction efficiency, selectivity and sustainability in a product- and condition-dependent manner. Solvent extraction remains relevant for selected aromatic products such as concretes and absolutes, while headspace techniques provide analytical tools for volatile profiling, quality control and comparison among extraction-derived products.

Nevertheless, direct comparison across studies remains constrained by heterogeneity in botanical materials, yield denominators, operating conditions, analytical workflows and biological assays, as well as by incomplete reporting of energy use, water demand, solvent or CO_2_ recovery and scale-up performance. Future research should prioritize authenticated, chemotype-defined material; harmonized reporting of extraction conditions and mass and energy balances; standardized GC–FID/GC–MS quality control and biological assay protocols; and further pilot- and industrial-scale studies assessing reproducibility, safety, cost and life-cycle performance. These advances would strengthen the translation of laboratory-scale findings into scalable, application-oriented and environmentally documented EO production.

## Figures and Tables

**Table 1:** Chemical-class-guided decision matrix linking EO constituent properties with extraction risks, method-selection considerations and analytical quality-control endpoints.

Target Class	Main Extraction Risks	Method-Selection Considerations	Analytical QC Endpoints
Monoterpene hydrocarbons	Volatilization losses, oxidation and isomerization during prolonged heating or storage	Short optimized SD/HD, MAHD/SFME or mechanical expression for citrus peels; avoid excessive extraction time	GC–FID/GC–MS profile, marker ratios and oxidation markers
Oxygenated monoterpenes	Hydrolysis, oxidation, thermal degradation or selective enrichment	Optimized SD/HD, MAHD or SFME; SFE-CO_2_ when volatile-rich fractions are targeted	Quantification of marker alcohols, aldehydes, ketones or oxides; sensory/authenticity ratios
Sesquiterpenes and oxygenated sesquiterpenes	Incomplete recovery during short distillation, thermal rearrangement and co-extraction of heavier lipophilic material	Optimized distillation, SFE-CO_2_ or solvent extraction depending on whether a classical EO or EO-related aromatic extract is targeted	Extended GC–MS/GC–FID profiling and target sesquiterpene quantification
Phenylpropanoids/ benzenoids	Thermal changes, oxidation, water partitioning and redistribution among EO, hydrolate, concrete or absolute	SD/HD for classical EOs; solvent extraction or SFE-CO_2_ for broader aromatic extracts; HS-SPME for native volatile profiling	Marker quantification, comparison of EO, hydrolate and EO-related aromatic extracts and headspace volatile profile

Abbreviations: EO, essential oil; GC–FID, gas chromatography–flame ionization detection; GC–MS, gas chromatography–mass spectrometry; SD, steam distillation; HD, hydrodistillation; MAHD, microwave-assisted hydrodistillation; SFME, solvent-free microwave extraction; SFE-CO_2_, supercritical CO_2_ extraction; HS-SPME, headspace solid-phase microextraction; QC, quality control.

**Table 2:** Qualitative comparative overview of essential-oil-related extraction, pretreatment and analytical approaches discussed in this review. The table summarizes the main advantages, limitations, sustainability-related aspects and typical applications of each approach, rather than providing a quantitative ranking of extraction performance.

Method	Main Advantages	Main Limitations	Sustainability-Related Aspects	Best Suited for	References
Steam distillation	Scalable, solvent-free and industrially established	Heat exposure, long extraction time and possible loss of thermolabile volatiles	Avoids organic solvents but may require substantial energy and water input	Robust EOs and industrial-scale production	[3,26,27]
Hydrodistillation	Simple, low-cost and widely standardized	Direct water contact, hydrolysis risk and thermal degradation	Useful benchmark method, but water and energy demand can be relevant	Laboratory comparisons and pharmacopeial/reference extraction	[3,39]
MAHD/SFME	Rapid extraction, reduced extraction time and often lower water and energy demand	Scale-up, microwave penetration and equipment constraints	Process intensification with potential reductions in extraction time, water and energy use	Moisture-rich matrices and rapid EO recovery	[28,40,41,42]
SFE-CO_2_	Mild temperature, tunable selectivity and organic-solvent-free CO_2_ extracts or volatile-rich fractions	High-pressure equipment, capital cost and possible co-extraction of non-volatile compounds	CO_2_ can be recycled and organic solvent residues are minimized, but equipment and pressure requirements are relevant	Lipophilic or thermolabile target compounds and high-value volatile-rich CO_2_ extracts	[39,43,44,45,46]
Solvent extraction	Effective for delicate flowers and low-volatility aromatic fractions	Solvent residues, wax co-extraction and non-equivalence to classical distilled EOs	Solvent choice, toxicity, recovery and waste management are critical	Concretes, absolutes and fragrance applications	[3,47,48]
UAE	Improves mass transfer and may reduce extraction time and solvent use	Reproducibility, acoustic-field distribution and scale-up constraints	Can support pretreatment, by-product valorization and reduced processing time	Pretreatment before hydrodistillation, EO recovery intensification and plant by-product valorization	[49,50,51,52,53]
Headspace/HS-SPME	Solvent-free analytical profiling, low sample requirement and useful aroma screening	Not a productive extraction method and does not provide EO yield data	Minimizes sample and solvent use in analytical screening	Quality control, volatile profiling and comparison of aroma fractions	[3,54,55,56]

Abbreviations: EO, essential oil; MAHD, microwave-assisted hydrodistillation; SFME, solvent-free microwave extraction; SFE-CO_2_, supercritical CO_2_ extraction; UAE, ultrasound-assisted extraction; HS-SPME, headspace solid-phase microextraction. Note: The approaches listed in the table differ in product boundary. Steam distillation, hydrodistillation and distillation-based or solvent-free microwave approaches used for EO isolation are considered classical EO recovery methods. Solvent extraction yields EO-related aromatic extracts, such as concretes, absolutes, resinoids or oleoresins, rather than classical distilled EOs. SFE-CO_2_ may produce CO_2_ extracts or volatile-rich fractions that should not automatically be considered equivalent to classical EOs. UAE is considered mainly as a pretreatment or intensification strategy in EO recovery, whereas headspace and HS-SPME are analytical volatile-profiling techniques and not productive extraction methods.

**Table 3:** Selected comparative studies showing the influence of extraction strategy on EO or EO-related product yield, volatile composition and process-related indicators. The table summarizes within-study comparisons discussed in Section 4 and illustrates method-dependent effects without aiming to provide an exhaustive systematic comparison or direct cross-study ranking.

Plant Matrix	Compared Methods	Reported Outcomes	References
*Salvia rosmarinus*	HD vs. MAHD	Comparable EO yield; extraction time reduced from 180 to 20 min; electric consumption reduced from 2.25 to 0.23 kWh; estimated CO_2_ emissions reduced from 1800 to 184 g; slight increase in oxygenated compounds with MAHD.	[40]
Coriander seeds	HD vs. MAHD	Similar EO yields, 0.31% and 0.325%; extraction time reduced from 240 to 60 min; energy consumption reduced from 2.52 to 0.63 kWh; linalool was the main constituent in both oils.	[41]
*Curcuma* spp.	HD vs. SFME	Major compounds were qualitatively similar, but quantitative profiles differed according to extraction method; HD oils were dominated by hydrocarbon compounds, whereas SFME oils were richer in oxygenated compounds; SFME required less time, energy and water and produced less waste.	[28]
*Citrus medica* peel	HD vs. SFE-CO_2_	SFE-CO_2_ gave higher extraction yield than HD, 7.60% vs. 4.50%, under milder thermal conditions, 35 °C/100 bar/30 min vs. 100 °C/1 bar/180 min. The SFE-CO_2_ extract showed a higher limonene percentage by GC–MS; higher colorimetric TPC/TFC values were also reported, but are interpreted here as assay-dependent functional indicators rather than direct evidence of volatile composition.	[39]
Citrus waste by-products	HD vs. ultrasound pretreatment + HD	Ultrasound pretreatment increased EO recovery by approximately 33% and reduced extraction time from 120 to 80 min.	[50]
*Rosa* × *damascena* fresh petals	SFME, MHG, HD and SD	Microwave-mediated and conventional extraction procedures produced different EO profiles; conventional EOs obtained by SD and HD were dominated by oxygenated terpenes, whereas microwave-mediated EOs obtained by MHG and SFME were dominated by alcohols.	[62]

Abbreviations: EO, essential oil; GC–MS, gas chromatography–mass spectrometry; HD, hydrodistillation; MAHD, microwave-assisted hydrodistillation; MHG, microwave hydrodiffusion and gravity; SD, steam distillation; SFE-CO_2_, supercritical CO_2_ extraction; SFME, solvent-free microwave extraction; TFC, total flavonoid content; TPC, total phenolic content.

## Data Availability

No new datasets were generated or analyzed during the preparation of this review. The information discussed in this article is derived from previously published studies, which are cited in the reference list.
